# Does elevated glucose metabolism correlate with higher cell density in Neurofibromatosis type 1 associated peripheral nerve sheath tumors?

**DOI:** 10.1371/journal.pone.0189093

**Published:** 2017-12-05

**Authors:** Dominik Berzaczy, Marius E. Mayerhoefer, Amedeo A. Azizi, Alexander R. Haug, Daniela Senn, Dietrich Beitzke, Michael Weber, Tatjana Traub-Weidinger

**Affiliations:** 1 Department of Biomedical Imaging and Image-guided Therapy, Division of General and Pediatric Radiology, Medical University of Vienna, Waehringer Guertel, Vienna, Austria, E.U; 2 Department of Pediatrics and Adolescent Medicine, Medical University of Vienna, Waehringer Guertel, Vienna, Austria, E.U; 3 Department of Biomedical Imaging and Image-guided Therapy, Division of Nuclear Medicine, Medical University of Vienna, Waehringer Guertel, Vienna, Austria, E.U; Northwestern University Feinberg School of Medicine, UNITED STATES

## Abstract

**Purpose:**

To investigate whether elevated glucose metabolism in neurofibroma, determined by [F18]-FDG-PET, is correlated with cell density in MRI, as expressed through the apparent diffusion coefficient.

**Materials and methods:**

Patients diagnosed with neurofibromatosis type 1 and peripheral nerve sheath tumors (PNST) were enrolled in this prospective, IRB-approved study. After a single [F18]-FDG injection, patients consecutively underwent [F18]-FDG-PET/CT and [F18]-FDG-PET/MRI on the same day. Maximum and mean standardized uptake values (SUVmax, SUVmean) on [F18]-FDG-PET/CT and [F18]-FDG-PET/MRI were compared, and correlated with minimum and mean apparent diffusion coefficients (ADCmean, ADCmin).

**Results:**

A total of 12 (6 male/6 female, mean age was 16.2 ± 5.2 years) patients were prospectively included and analyzed on a per-lesion (n = 39) basis. The SUVmean of examined PNST showed a moderate negative correlation with the ADCmean (r = -.441) and ADCmin (r = -.477), which proved to be statistically significant (p = .005 and p = .002). The SUVmax of the respective lesions, however, showed a weaker negative correlation for ADCmean (r: -.311) and ADCmin (r: -.300) and did not reach statistical significance (p = .054 and p = .057).

Lesion-based correlation between [F18]-FDG-PET/MRI and [F18]-FDG-PET/CT showed a moderate correlation for SUVmax (r = .353; p = .027) and a strong one for SUVmean (r = .879; p = .001)). Patient-based liver uptake (SUVmax and mean) of [F18]-FDG-PET/MRI and [F18]-FDG-PET/CT were strongly positively correlated (r = .827; p < .001 and r = .721; p < .001) but differed significantly (p < .001).

**Conclusions:**

We found a statistically significant, negative correlation between glucose metabolism and cell density in PNST. Thus, ADCmean and ADCmin could possibly add complimentary information to the SUVmax and SUVmean and may serve as a potential determinant of malignant transformation of PNST.

## Introduction

Neurofibromatosis type 1 (NF1) is an autosomal dominant neurocutaneous disorder caused by a mutation of the tumor suppressor gene NF1 (17q11.2) with a rate of approximately 1 in 3000 live births [1–3]. NF1 is a tumor pre-disposition syndrome, i.e. patients carry a high risk of developing benign and malignant tumors [4–6]. Diagnosis is made genetically or clinically using the NIH criteria which are based on typical features such as Café au lait macules, neurofibroma, optic pathway glioma, iris hamartoma (Lisch nodules) and osseous dysplasia [7].

Neurofibroma most commonly show cutaneous or subcutaneous, focal dermal growth or can be located adjacent to the spine or intraforaminal and may appear as nodular or diffuse plexiform neurofibroma. The latter are of particular importance as they have an increased risk of malignant transformation with a lifetime risk that has been reported from 5 to 13% [8,9].

These malignant peripheral nerve sheath tumors (MPNST) are difficult to detect as malignant transformed tissue is often embedded within benign lesions [10].

When present, MPNST show a poor prognosis with an overall 5-year-survival of about 42% due to delayed diagnosis, early metastasis (liver, lung, brain, lymph nodes and bones) and limited response to systematic therapy [3,5,8,9,11].

Patients with NF1 therefore demand increased surveillance. Surgical resection is technically challenging due to the anatomic location of these tumors, not only as the mainstay of therapy, but also when obtaining a histological specimen for diagnostic purposes. Morphologic imaging parameters of these tumors obtained by contrast-enhanced computed tomography (CE-CT) and magnetic resonance imaging (MRI) cannot determine malignant transformation to a satisfactory extent. Repeated biopsies, on the other hand, are uncommon in the clinic and obviously, no acceptable option. Nowadays, [F18]fluoro-2-desoxy-D-glucose-positron emission tomography ([F18]-FDGPET) imaging is in use to help differentiate between benign and malignant PNST in patients with NF 1. To the best of our knowledge, no data have been published concerning a correlation between the standardized uptake value (SUV) and apparent diffusion coefficient (ADC) in peripheral nerve sheath tumors in the setting of NF 1 obtained simultaneously using [F18]-FDG-PET/MRI.

Purpose of this report was to investigate whether glucose metabolism in plexiform neurofibroma, determined by [F18]-FDG-PET, is correlated with cell density on diffusion-weighted MRI (DWI), as expressed through the ADC, which may represent a complimentary imaging-derived biomarker to detect MPNST.

## Materials and methods

### Patient population and study design

Pediatric, adolescent and young adult patients diagnosed with neurofibromatosis type 1 and peripheral nerve sheath tumors, were enrolled in this prospective, Institutional Review Board (ethics committee of the medical university of vienna) approved, dual-modality study. Written informed consent was obtained from all adult patients and, in case of minor participants, from their guardians, prior to imaging. After a single [F18]-FDG injection, patients consecutively underwent [F18]-FDG-PET/CT and [F18]-FDG-PET/MRI on the same day, for initial staging or follow-up purposes. General contraindications to MRI (e.g., severe claustrophobia, unsafe metal implants for a field strength of 3T), elevated glucose levels (>150mg/dl), pregnancy and known adverse reactions to ionized contrast media served as exclusion criteria.

### Imaging protocols

[F18]-FDG-PET/CT and [F18]-FDG-PET/MRI were performed consecutively on the same day, using only a single injection of [F18]-FDG for both examinations.

[F18]-FDG-PET/CT was generally performed first, covering the anatomy from head to toe, using a 64-row multi-detector hybrid PET/CT device (Biograph TruePoint 64; Siemens, Erlangen, Germany). For PET, this scanner offers an axial field-of-view of 216 mm, a sensitivity of 7.6 cps/kBq, and a transaxial resolution of 4–5 mm. After patients had fasted for five hours, PET was performed 45–60 min after an intravenous administration of a mean of 225 ± 64MBq of [F18]-FDG, with a 3-min/bed position, four iterations, and 21 subsets, a 5-mm slice thickness, and a168x168 matrix, using the point-spread function (PSF)-based reconstruction algorithm TrueX. Venous-phase CE-CT was used for attenuation correction, and was obtained after the intravenous injection of a mean of 74 ml of a tri-iodinated, non-ionic contrast medium (adjusted to body weight) at a rate of 2 ml/s; a tube current of 120 mA; a tube voltage of 230kV; a collimation of 24x1.2 mm; a 3-mm slice thickness with a 2-mm increment; and a 512x512 matrix. In 4 cases a low dose CT protocol was applied using 120Kv and 50mAs without any contrast media.

[F18]-FDG-PET/MRI, covering the anatomy from the vertex to the upper thigh was performed directly after PET/CT, using an integrated, simultaneous, hybrid PET/MRI device (Biograph mMR; Siemens, Erlangen, Germany) operating at 3 Tesla, with high-performance gradient systems (45 mT/m) and a slew rate of 200 T/m/s, and equipped with a phased-array body coil.

For PET, the system offers an axial FOV of 256 mm, a sensitivity of 13.2 cps/kBq, and a transaxial resolution of 4.4 mm. PET was performed 100–150 min after the original tracer administration, with a 5-min/bed position, three iterations, and 21 subsets, a 4.2-mm slice thickness, and a 172x172 matrix, using the point spread function-based reconstruction algorithm HD-PET.

A coronal T1-weighted (T1w) two-point DIXON breath-hold MR sequence (VIBE) was acquired for attenuation correction, using the following parameters: repetition time (TR)/echo times (TE) 3.6/TE1 = 1.23 ms TE2 = 2.46 ms; one average, two echoes; a 10° flip angle; a 320x175 matrix with a 430x309 mm FOV; and a 3-mm slice thickness with 0.6-mm gap.

For anatomic correlation an axial VIBE, with the following parameters was used: repetition time (TR)/echo times (TE), 4.9/2.1ms; one average, a 9° flip angle; a 172x320 matrix with a 273x380 mm FOV; and a 3-mm slice thickness with 0.6-mm gap.

A single-shot, echo-planar imaging (EPI)-based, spectral adiabatic inversion recovery (SPAIR) diffusion-weighted imaging (DWI) sequence was obtained with the following parameters: b-values, 50 and 800; TR/TE, 6600/63 ms; six averages and one echo; a 180° flip angle; a 168x104 matrix with a 440x340 mm FOV; and a 6-mm slice thickness with a 1.2-mm gap.

### Qualitative and quantitative image analysis

A board-certified radiologist and a board-certified nuclear medicine physician performed all image analyses in consensus (side-by-side reading) to guarantee that the same lesions were chosen for the quantitative analysis on PET and DWI.

In a first step, to correlate FDG PET and DWI of PETMRI, a maximum of 5 PNST lesions per patient, localized adjacent to nerve fascicles that arise from major nerve branches, that had to be visible on T1w and DWI with a 2cm minimum long axis diameter were defined. Their corresponding maximum and mean standardized uptake values (SUVmax and mean) were measured on [F18]-FDG-PET based on isocontour volumes of interest (VOIs) that included all voxels above 50% of the SUVmax of each lesion, and were constructed using the Syngo MultiModality Workplace environment (Siemens, Erlangen, Germany). Similarly, minimum and mean apparent diffusion coefficients (ADCmin and ADCmean, (x10^-6^ mm^2^/s)) of respective lesions were measured on ADC maps, using the interactive level-set method of the MIPAV 7.2.0 software package (Center for Information Technology, NIH, USA).

Secondly, solely for comparison of the PET components of PETCT and PETMR, the SUV values of the largest lesion visible both on CT and T1w images in PETMR, as well as the SUVs of the liver parenchyma were measured by placing spherical volumes of interest (VOIs) with a 2-cm diameter over the lesion and over a lesion-free part of the liver parenchyma, respectively.

### Statistical analysis

Pearson correlation coefficients (r) were used to assess the relationship between liver parenchymal and lesional uptake in [F18]-FDG-PET/MRI and [F18]-FDG-PET/CT, and between the imaging-derived parameters SUVmax and SUVmean and the respective ADC values, on a per-lesion basis, on [F18]-FDG-PET/MRI. Continuous variables are expressed as arithmetic mean ± standard deviation (SD). Categorical variables are presented as absolute frequencies. Paired t-tests were performed to compare SUVmax and SUVmean values measured on PET/MRI and PET/CT, respectively. The level of significance was set to p ≤.05 for all tests. Statistical tests were performed using IBM SPSS Statistics 21.0 software (IBM Corp., Armonk, NY, USA).

## Results

### Patients

A total of twelve patients–six males and six females–with a total of 39 lesions were prospectively included between January 2014 and June 2015. The mean age was 16.2 ± 5.2 (SD) years.

### Correlation of SUV and ADC

These calculations were performed on a per-lesion basis (up to five different PNST loci per patient).

The SUVmean showed a statistical significant moderate negative correlation with the ADCmean (r = -.441, p = .005) and ADCmin (r = -.477, p = .002).

**Fig 1:**
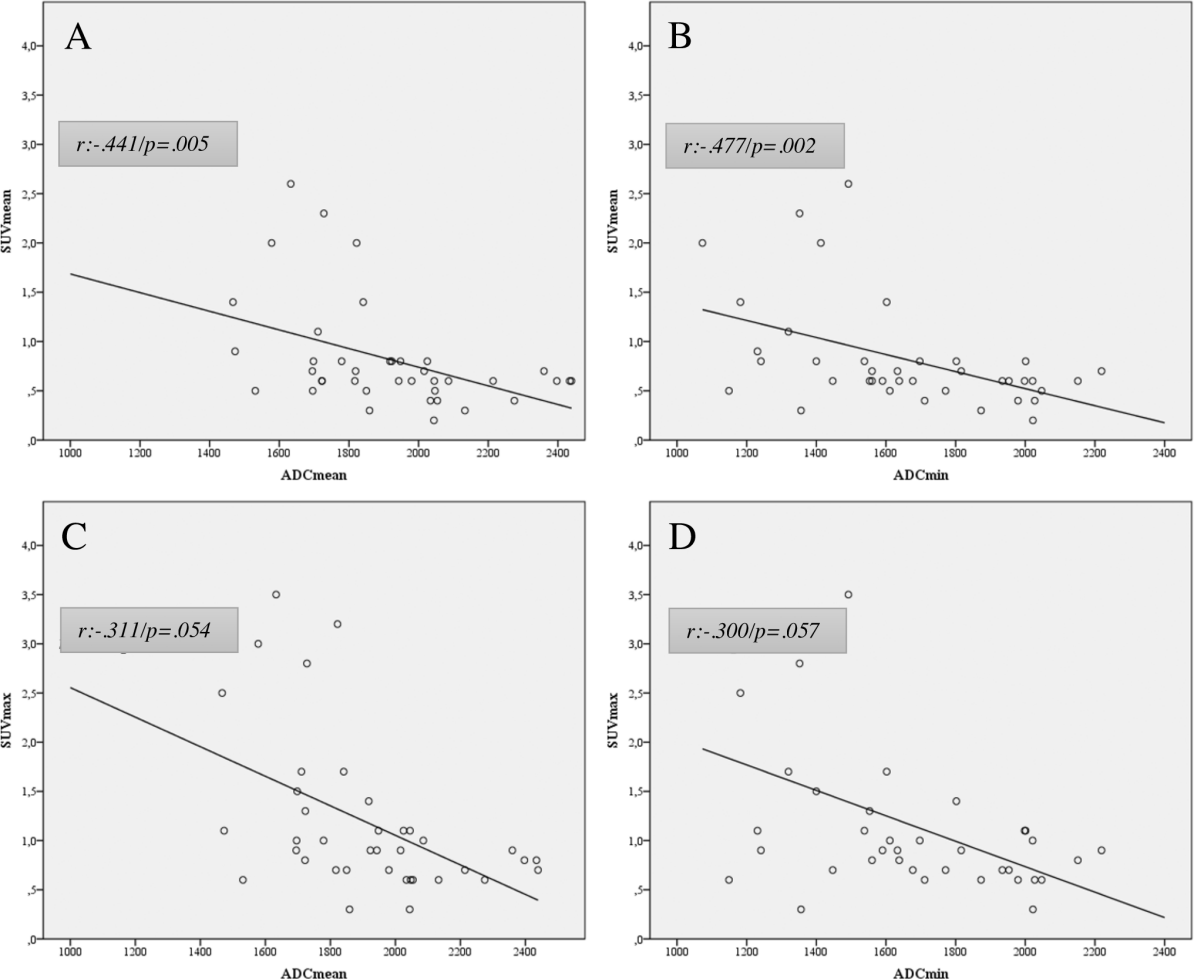
Correlation of glucose metabolism and cell density in PET/MRI. (A and B) SUVmean showed a moderate, inverse correlation to ADC mean and min. (C and D) SUVmax showed a weak inverse correlation for and ADC mean and min.

However, the SUVmax of the respective lesions showed a weaker negative correlation for ADCmean (r = -.311, p = .054) and ADCmin (r = -.300, p = .057), and only showed a trend towards statistical significance (Fig 1).

### Correlation of the PET components of [F18]-FDG-PET/MRI and [F18]-FDG-PET/CT

SUVmean showed a strong, statistical significant correlation (r = .879; p = .001) as SUVmax showed a moderate, also statistical significant correlation (r = .353; p = .027) between the PET components of both techniques.

**Fig 2:**
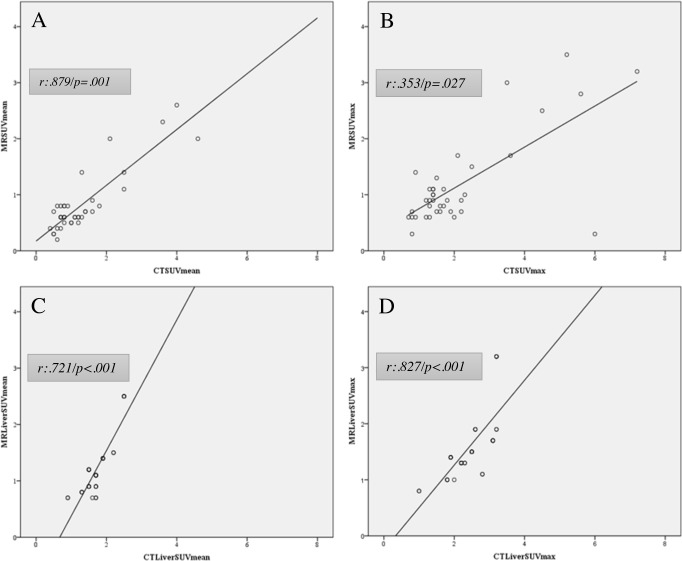
Correlation of the PET component of PET/MRI and PET/CT. (A) SUVmean showed a strong positive correlation and (B) SUVmax a moderate positive correlation for PET/MRI and PET/CT. (C and D) SUV mean and max showed a strong positive correlation for patient-based liver uptake.

Patient-based liver uptake (SUVmax and SUVmean) of [F18]-FDG-PET/MRI and [F18]-FDG-PET/CT was found to be strongly positively correlated (r = .827; p < .001; and r = .721; p < .001) but differed significantly for both SUVmax and SUVmean (p < .001) (Fig 2).

## Discussion

Determination of malignant transformation in peripheral nerve sheath tumors is a challenging task on many levels. The use of either clinical symptoms, that suggest malignancy (e.g. neurological deficits, fast increasing tumor volume, new onset pain lasting for more than four weeks) or imaging modalities only assessing morphologic parameters provide only very limited ability to characterize these tumors and may not be specific enough to differentiate between benign and malignant peripheral nerve sheath tumors (PNSTs). MPNST are highly aggressive tumors that represent a frequent cause of death in these patients [12]. Hence, theses tumors warrant early diagnosis to allow early, potential curative therapy. Therefore, non-invasive diagnostic imaging methods are highly desirable.

Today, [F18]-FDG-PET/CT is a well-established imaging modality, which is most commonly used in clinical routine in staging, restaging, therapy monitoring and to help identifying possible MPNSTs in symptomatic NF1 patients, offering a high sensitivity [13].

[F18]-FDG-PET imaging allows a semi-quantitative measure of glucose metabolism and calculation of SUV and its derived parameters that reflect increased metabolic activity and levels of overexpression of its respective receptors. However, the crucial point of identification of malignant transformation by using a distinctive SUVmax as cutoff to differentiate between benign and malignant plexiform neurofibroma remains not fully clear and recent literature reports varying results regarding [F18]-FDG imaging [3,6].

Literature reports optimal cut-off values, with regard to maximized sensitivity and specificity, ranging from 3.1 to 6.1 [13–17].

Tovmassian et al. [3] report in a recent review, that most of proposed cut-off values for SUVmax are found between 3 and 4, which is a range in that both benign and malignant PNSTs can occur and hence makes clinical decision making difficult. Furthermore, the SUVmax can be influenced by multiple factors arising from the use of different scanners, variability in technical parameters, and image reconstruction and calibration [18]. However, a SUVmax > 3.5 is commonly used in clinical practice and research in this regard.

Azizi et al. [19] recently published a study comprising data on symptomatic and asymptomatic NF1 patients showing the value of FDG PET in the work-up of NF1 patients.

Promising approaches to investigate alternative or complimentary imaging derived markers to SUVmax have been published recently. Salamon et al. [20] reported an increased specificity using a SUVmax tumor-to-liver (TTL) ratio of 90.3% in attempt to overcome the variability of the SUVmax. The same group published data showing a statistically significant higher rate of metabolic tumor volume (MTV) and total lesion glycolysis (TLG) in MPNSTs. In support of this data Van der Gucht et al. [21] could show the utility of TLG as prognostic marker for survival when compared to TTL and the SUVmax. Chirindel et al. [22] also showed good clinical utility of [F18]-FDG-PET when using lesion to liver activity normalization when comparing early and delayed imaging which yielded similar accuracy in qualitative interpretation of neurofibroma. However, SUVmax, despite its limitations, seems to be the most useful parameter in [F18]-FDG-PET based on available data in literature [3].

With the advent of hybrid PET/MRI scanners, a hybrid imaging method that combines metabolic PET information and DWI in a single examination became available for clinical practice. In this preliminary study we investigated whether (semi)-quantitative measures of SUVs and ADCs derived from [F18]-FDG-PET and DWI are correlated. As sarcomatous tissue shows an increased glucose metabolism that is believed to be linked to a higher cell density and lower differentiation grade, a correlation of both parameters seems a valid approach. Furthermore, we investigated how the PET components of [F18]-FDG-PET/CT and [F18]-FDG-PET/MRI correlate to provide connecting data on both imaging methods in these consecutively performed examinations.

**Fig 3:**
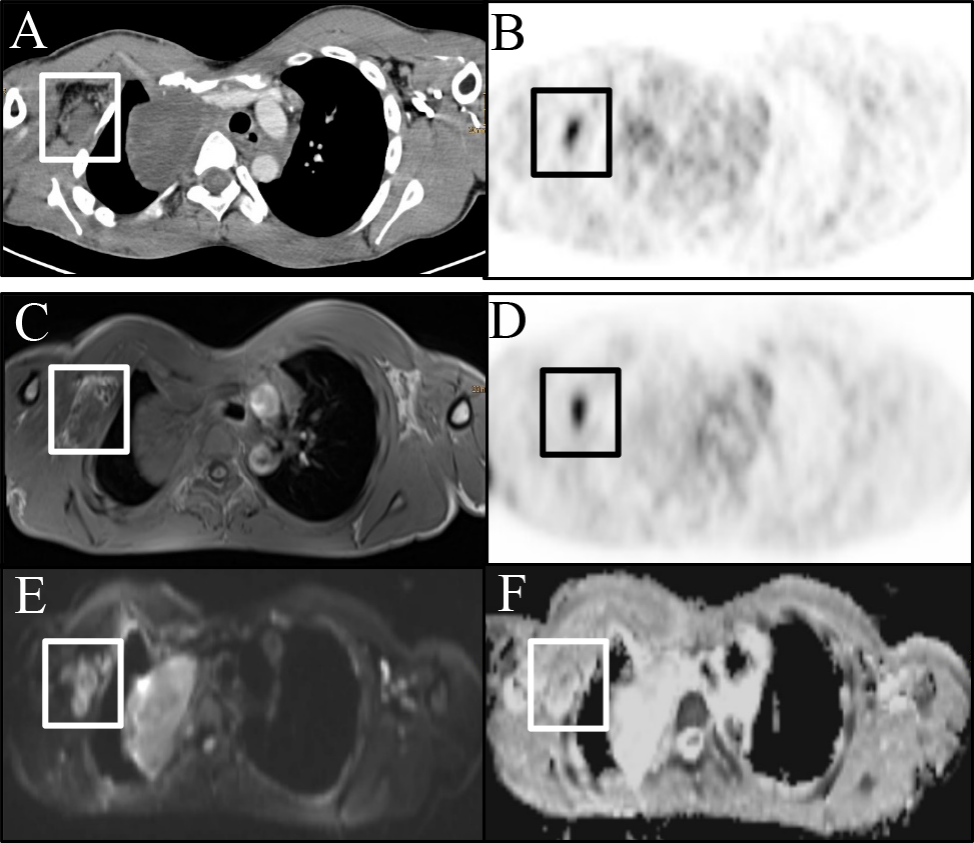
PNST in a 17-year male patient. (A) Peripheral nerve sheath tumor depicted on CE-CT image with (B) increased glucose metabolism. (C) Corresponding T1w image. (D) [18F]-FDG-PET depicting increased glucose uptake and corresponding restricted diffusion on (E) DWI (b800) image with (F) lowered signal on the ADC map.

**Fig 4:**
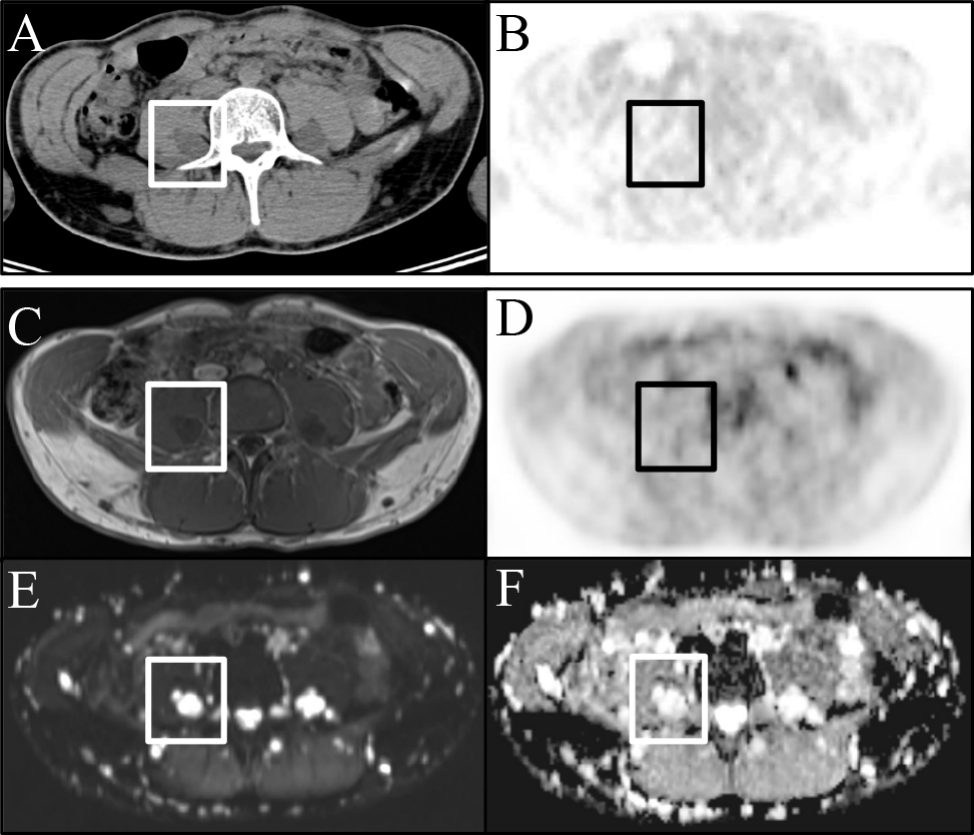
PNST in a 22 -year male patient. (A) CE-CT image and corresponding (B) PET image with no pathological glucose uptake. (C) T1w image and corresponding (D) PET images with also no abnormal focal tracer uptake. (E) DWI (b800) and (F) ADC maps showing restricted diffusion but high signal on the ADC map.

As our main result we found a moderate, statistically significant negative correlation of the SUVmean of examined peripheral nerve sheath tumors with the ADCmean and ADCmin (Figs 3 and 4).

These results are comparable to published findings of inverse correlation between SUVs and ADCs that have been reported in varying degrees and in different tumor entities. Schaarschmidt et al. found a significant inverse correlation in metastatic lymph nodes in NSCLC between SUVmean and ADCmean as well as between SUVmax and ADCmean [23].

Other authors reported similar results with strong inverse correlations between SUVmax and ADCmin in primary cervical cancer [24], and a work by Rakheja et al. [25], comprising different malignant tumors. ADC values that, similar to the SUVmax, carry some variability depending on manufacturer and field strength, seem to offer an additional value in monitoring therapy response as they have shown superiority over SUVmax in studies including NSCLC and MALT lymphoma [26,27].

Interestingly, our results show that the SUVmax of NF1 associated nerve sheath tumors showed a rather weak, negative correlation with ADCmean and ADCmin that only showed a clear trend towards statistical significance; however, it has to be acknowledged that our sample size was possibly too small to draw definitive conclusions. Our results are in good accordance with the published literature, especially for the SUVmean, which seems advantageous to us due to its robust nature.

Comparing the PET component of [F18]-FDG-PET/MRI to [F18]-FDG -PET/CT we found a positive correlation for SUVmax and mean for the per-patient lesion and liver uptake which differed statistical significantly between the two methods. These differences are most of all due to technical differences in scatter and attenuation correction as well as different distribution of respective tracer.

Our preliminary study has limitations, the most obvious of which is the small sample size that prevented us from performing a subgroup evaluation with regard to pathohistology on a per-lesion basis. Although we found significant correlations, this fact did not allow a direct comparison between the performances of [F18]-FDG-PET and DWI.

In conclusion, we found a statistically significant negative correlation between the SUVmean and the ADCmean as well as between the SUVmean and the ADCmin in peripheral nerve sheath tumors in NF1 patients. Based on our findings we believe that a combination of the two techniques, preferably combined in the form of [F18]-FDG-PET/MRI, would possibly offer complimentary information to help characterizing PNSTs. The simultaneous acquisition of SUVs and ADCs is a good compromise between examination time and patient comfort and offers advantages in terms of clinical workflow optimization. Nevertheless, it seems obvious that discrimination between malignant and benign PNSTs and consecutive clinical decision making in NF1 patients should be based on a holistic patient-based approach, taking all available, noninvasive methods and its parameters into account. Our findings suggest that [18F]-FDG-PET and DWI offer complimentary information which might increase diagnostic accuracy, but larger cohorts to support current preliminary data are needed.

## Supporting information

S1 File Original data of the present study. (XLSX)
